# DNA- and DNA-Protein-Crosslink Repair in Plants

**DOI:** 10.3390/ijms20174304

**Published:** 2019-09-03

**Authors:** Janina Enderle, Annika Dorn, Holger Puchta

**Affiliations:** Botanical Institute, Molecular Biology and Biochemistry, Karlsruhe Institute of Technology, Fritz-Haber-Weg 4, 76131 Karlsruhe, Germany

**Keywords:** crosslink repair, DPC, ICL, intrastrand CL, Fanconi Anemia, helicase, protease

## Abstract

DNA-crosslinks are one of the most severe types of DNA lesions. Crosslinks (CLs) can be subdivided into DNA-intrastrand CLs, DNA-interstrand CLs (ICLs) and DNA-protein crosslinks (DPCs), and arise by various exogenous and endogenous sources. If left unrepaired before the cell enters S-phase, ICLs and DPCs pose a major threat to genomic integrity by blocking replication. In order to prevent the collapse of replication forks and impairment of cell division, complex repair pathways have emerged. In mammals, ICLs are repaired by the so-called Fanconi anemia (FA) pathway, which includes 22 different *FANC* genes, while in plants only a few of these genes are conserved. In this context, two pathways of ICL repair have been defined, each requiring the interaction of a helicase (FANCJB/RTEL1) and a nuclease (FAN1/MUS81). Moreover, homologous recombination (HR) as well as postreplicative repair factors are also involved. Although DPCs possess a comparable toxic potential to cells, it has only recently been shown that at least three parallel pathways for DPC repair exist in plants, defined by the protease WSS1A, the endonuclease MUS81 and tyrosyl-DNA phosphodiesterase 1 (TDP1). The importance of crosslink repair processes are highlighted by the fact that deficiencies in the respective pathways are associated with diverse hereditary disorders.

## 1. Introduction

As sessile organisms, plants lack a strategy of damage avoidance and therefore are particularly exposed to harmful environmental influences. As a consequence of DNA lesions that are induced by a wide range of damaging factors, a great variety of DNA repair mechanisms have evolved in order to maintain genomic integrity. Covalent linkages within DNA strands, or between DNA and proteins, possess a high cytotoxic potential, and it remains a main challenge for cells to overcome these threats. As intrastrand crosslinks (CLs) only affect one DNA strand, they can be repaired more easily than interstrand CLs (ICLs) that connect and, therefore, compromise both complementary DNA strands. Similar to ICLs, DNA-protein crosslinks (DPCs) exhibit a physical obstacle to the replication machinery, mandatorily requiring efficient and fast-acting repair mechanisms in order to prevent blocking of the replication fork. In this review, we sum up the formation of the distinct types of CLs, the consequences of unrepaired CLs and the current knowledge of CL repair mechanisms in plants and other organisms.

## 2. Sources of DNA Crosslinks

**Figure 1:**
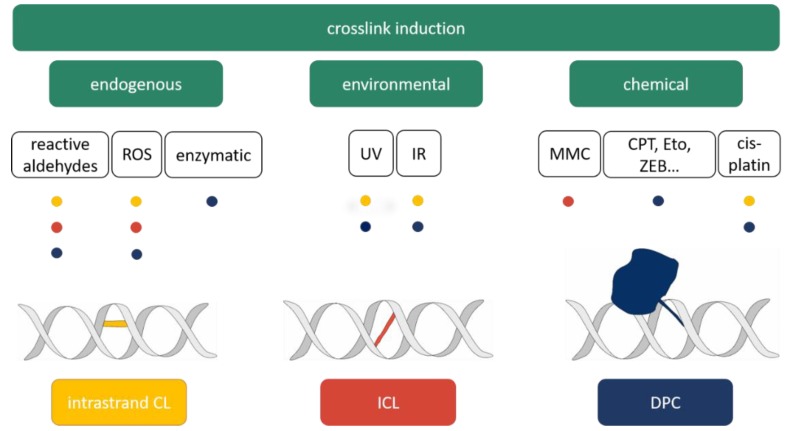
Overview on the origin of different types of crosslinks. The different origins of CL induction by endogenous, environmental and chemical factors are summarized. Reactive aldehydes, reactive oxygen species (ROSs) and stabilization of enzymatic reaction intermediates are able to endogenously produce CLs. UV and ionizing radiation (IR) are environmental CL sources. Chemical crosslinkers form the third category, including mitomycin C (MMC), camptothecin (CPT), etoposide (Eto), zebularine (ZEB) and cis-platin. The colored dots provide information on the type of induced CL. Intrastrand CL: yellow; ICL: orange; DPC: blue.

CLs represent a severe threat to genomic integrity and can be caused by a wide range of endogenous, environmental and chemical factors. Figure 1 provides an overview of CL-inducing sources and the resulting types of lesions.

Reactive aldehydes—such as formaldehyde, which is produced during the demethylation of histones [1,2,3], or acetaldehyde, which results from ethanol metabolism or as an intermediate of sugar metabolism [4]—are able to endogenously induce all three types of CLs: intrastrand CLs, ICLs and DPCs. For CL induction, the nucleophilic primary amine of a DNA base and the carbonyl carbon of an aldehyde form a methylol adduct that is subsequently converted to a Schiff base. In case of another primary amine of a DNA base in close proximity, intrastrand CLs or ICLs can be formed. Reaction with a lysine or arginine residue of a protein, in contrast, leads to the formation of a covalent linkage between protein and DNA, thus producing a DPC [4,5,6,7,8,9].

Reactive oxygen species (ROSs) arise from various metabolic processes in the cell. In plants, ROSs are of particular importance as they are produced during a fundamental plant-specific process: photosynthesis. In this context, ROSs result from the side reactions of involved oxidases [10,11]. In mechanisms of plant pathogen defense, however, ROSs are exploited in order to kill pathogens and pathogen-infected plant cells [12,13,14].

In general, ROSs arise as a byproduct of molecular oxygen reduction. The result is the formation of the superoxide anion (O_2_^−^) from which other ROSs like H_2_O_2_ are derived. H_2_O_2_ can be further converted into water, and a hydroxyl radical (˙OH) that is strongly reactive and based on its strong electronegativity triggers a chain reaction of radical formation [15,16]. Interstrand crosslinking by ROSs mainly derives from C4′-oxidized abasic sites and nucleophilic addition to guanine radical cations [17]. DPCs can moreover indirectly arise from ROSs via the formation of apyrimidinic/apurinic (AP) sites, leading to covalent linkages of nearby proteins [6,18]. Apart from ICLs and DPCs, oxidative DNA damage further includes intrastrand CLs by bonding a nucleobase with the 5′ carbon of the 2-deoxyribose from the same nucleobase or a neighboring pyrimidine base [19,20].

Spontaneously occurring enzymatic DPCs are another endogenous source of DNA-protein adducts. In this scenario, technically reversible enzymatic reaction intermediates are trapped at the DNA, and subsequently persist as permanent covalent adducts. Predominantly, DNA processing enzymes such as type 1 and 2 topoisomerases or DNA-methyltransferases are subject to the formation of enzymatic DPC lesions [21,22,23,24]. In cases of a trapped TOP1 or TOP2, the resulting outcomes are referred to as topoisomerase 1 cleavage complexes (TOP1ccs) or topoisomerase 2 cleavage complexes (TOP2ccs). The key feature of this kind of enzymatic DPCs is the tyrosyl-phosphodiester bond that is stabilized between the DNA backbone and the protein [25].

Environmental influences such as UV and ionizing radiation (IR) represent a second class of CL-inducing factors. Both types of radiation are able to contribute to DNA damage through the introduction of intrastrand CLs and DPCs. The most well-known intrastrand CLs caused by UV radiation are pyrimidine pyrimidone 6-4 photoproducts and cyclobutane pyrimidine dimers [26,27,28]. Moreover UV and IR are able to induce DPCs, mostly resulting in the protein linked to an undisrupted DNA strand [6,29,30,31,32].

For research aiming to elucidate the repair of distinct types of CLs or for cancer treatment, chemical crosslinkers are frequently applied. The cytotoxic antibiotic mitomycin C (MMC), which is obtained from *Streptomyces caespitosus,* induces ICLs as the main adduct [33]. The activation of this substance occurs due to its reduction to an alkylant in the cell, which enables the linkage between complementary DNA strands [34].

Camptothecin (CPT), etoposide (Eto) and zebularine (ZEB) are compounds that are widely used to induce DPCs. CPT specifically targets topoisomerase 1. Topoisomerases are enzymes crucially needed to ensure the relaxation of the DNA after torsional tension. They function via the active tyrosine residue in the active center of the enzyme to attack the phosphate of the DNA backbone. This way, a tyrosyl-phosphodiester bond is formed, while simultaneously nicking the DNA backbone. After the supercoiling is resolved, the reverse reaction takes place, resulting in the religation of the backbone and the topoisomerase dissociates from the DNA [35]. CPT leads to the stabilization of the tyrosyl-phosphodiester bond by preventing the religation of the DNA backbone after topoisomerase 1 action, inducing TOP1ccs, which represent a specific type of enzymatic DPC accompanied by a single-strand break [21,36]. Eto induces TOP2ccs by trapping topoisomerase 2 at the DNA in a similar manner as described for CPT in the case of TOP1ccs [37,38,39]. Zebularine is a nucleoside analogous of cytidine that enables the covalent trapping of DNA methyltransferase (DNMT) after being incorporated in genomic DNA. Covalent adducts of DNMTs at the DNA are a further type of DPC, also known as nucleoprotein adducts (NPAs) [40].

Cis-diamin-dichloro-platin (II) (cis-platin) is one of the most broadly applied cytotoxic agents in cancer treatment for which a strong antitumor activity was proven in 1970 [41]. The effect of cis-platin is based on the induction of different DNA adducts, such as intrastrand crosslinks or DPCs. Ninety percent of all DNA lesions induced by cis-platin crosslinking occurs by binding the active cationic form of the N7 position of two purine bases [42,43]. In addition to intrastrand CLs (85–90%) [44], cis-platin is also able to induce DPCs (8–10%) [45] that can be described as ternary DNA-platin-protein adducts [46,47,48,49,50]. Here, cis-platin connects N7 positions of guanines with lysine, cysteine, histidine, glutamine or arginine residues of the protein. Using mass spectrometry approaches, more than 250 different proteins haven been identified as targets of cis-platin-induced crosslinking to the DNA [45]. It is important to note that not necessarily the frequency but also the nature of the respective lesion is an important determinant of the cytotoxicity of cis-platin.

## 3. Biological Consequences

Faithful duplication of DNA in S-phase is dependent on the reliable function of the replisome. Replisomes are multiprotein molecular machines that coordinate all crucial enzyme activities needed for replication [51,52,53]. Among a wide variety of replication-associated factors, replicative helicases and polymerases represent the key enzymes of this process.

**Figure 2:**
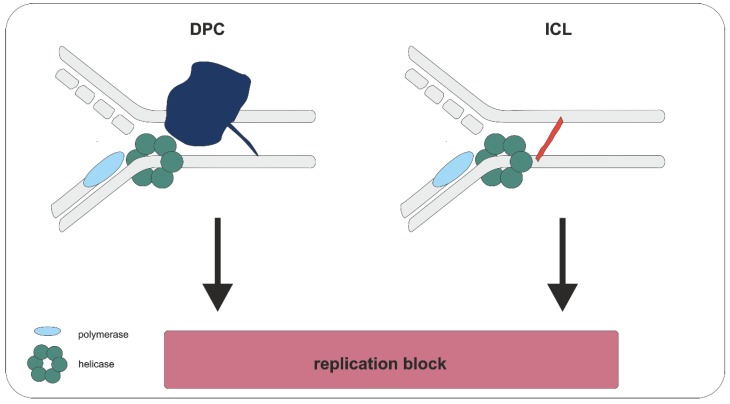
Blocking of replication by crosslinks. Crosslinks, like DPCs (blue) and ICLs (red) represent a physical obstacle for the replication machinery, including replication-associated key enzymes like helicases and polymerases. If left unrepaired, inhibition of replication compromises cell division and can consequently result in cell death.

ICLs and DPCs pose a great risk for living cells, as they block a variety of DNA metabolic processes such as replication and transcription by forming a physical obstacle [6,54,55,56]. While in the case of intrastrand CLs the complementary DNA strands can be separated and the intact strand can serve as template for repair, proper strand separation is compromised at the ICL and DPC sites. Here, the progression of key enzymes such as replicative helicases and polymerases is blocked (Figure 2).

In the case of DPCs, the biological consequences differ depending on their position. If the DNA-protein adduct is located on the leading strand, progression of the replicative helicase and polymerase is blocked. In vitro studies using DPC-mimicking biotin-streptavidin adducts showed that DNA unwinding is disturbed during replication [54,57,58], obstructing the replication fork progression in vivo [54,59]. DPCs located on the lagging strand do not interfere with helicase progression, but impede the translocation of the replicative polymerase [49,54,56,57,58,60,61].

ICLs affect both complementary DNA strands and thereby lead to an arrest of both the helicases and polymerases [54,62,63]. Inhibition of replication can result in genome instability and in blocking the cell division, leading to untimely cell death [32]. Therefore, it is not surprising that mutations in many of the known genes involved in CL repair are associated with severe human diseases.

Besides replication, crosslinks also block transcription. Here, the CLs are a barrier for proper RNA polymerase progression, thereby inhibiting the production of RNA transcripts that are crucially needed as templates for protein biosynthesis [54,64,65,66].

Taken together, CLs threaten cellular integrity at various levels and in numerous genetic processes. This major impact of crosslinks on cell viability is widely exploited in cancer treatment.

## 4. Repair of DPCs

Surprisingly, even though ICLs and DPCs are of comparable toxicity for cells and end in the same cell fate, detailed research on DPC repair was neglected for a long time. It has only been during the last few years that the central mechanism of repair of DPCs was elucidated.

DPCs represent a class of structurally highly diverse DNA adducts [54], and therefore many specific repair pathways have evolved. Analysis of non-enzymatic DPC repair first focused on the contribution of canonical DNA repair pathways such as nucleotide excision repair (NER) and homologous recombination (HR) [67,68]. It has been shown that NER is able to protect cells from DPC-inducing agents in bacteria and yeast [69,70,71], removing the majority of formaldehyde-induced DPCs before S-phase [69,72]. However, repair of DPCs via NER appear to be limited by the size of the covalently attached proteins. Protein adducts larger than 11 kDa are able to escape from NER-based repair [69,70,73]. The application of proteasome inhibitors impairs cells during DPC repair, suggesting the possibility that proteolytic activity can make bigger DPCs accessible too [9,73]. In *E.coli* it has been shown that DPCs can alternatively be repaired via RecBCD-dependent HR. Hypersensitivity of HR-deficient cell lines after treatment with DPC-inducing agents showed that HR also appears to be involved in DPC tolerance and repair in eukaryotes [69,70,74,75,76]. As DPCs do not only vary by the covalently bound protein, but also by the type of DNA structure involved, this serves as an additional feature for specialized repair. Type 2 topoisomerases, for example, lead to the formation of DPCs adjacent to a double-strand break (DSB). Thus, enzymes involved in DSB repair, such as the multifunctional MRN complex, can contribute to DPC repair. This has been proven by a distinct sensitivity of Mre11-deficient yeast cells after treatment with topoisomerase mutagens [77] and the repair of stabilized TOP2ccs via the conserved MR complex of T4 bacteriophages [78,79]. Replication fork regression could be one mechanism of DPC tolerance [80]. In such a case, the replication machinery could use the newly synthesized undamaged daughter strand as template, while the damage on the parental strand would remain. CPT-sensitive mutants of the RecQ-homologs ScSgs1 and HsBLM hint at an involvement of these helicases in the repair of CPT-induced lesions [81,82] where they could induce the regression of the replication fork. In Arabidopsis, topoisomerase 3α— acting in the RecQ-helicase associated RTR complex—could additionally be linked to DPC repair, as respective mutants exhibit a hypersensitivity to CPT [83].

An important pathway for the repair of stabilized TOP1cc is mediated by the enzymatic hydrolysis of the phosphodiester bond via tyrosyl-DNA phosphodiesterase 1 (TDP1). The specialized enzyme TDP1 resolves the phosphodiester bonds between the 3′-phosphate of the DNA backbone and the active tyrosyl residue of topoisomerase 1 [84,85]. *TDP1* is an strongly conserved gene (in evolutionary terms) that exists in all eukaryotic organisms, and its mutations lead to a hypersensitivity towards TOP1 inhibitors [37,84,85]. The activity of TDP1 is based on its HKN motifs, forming the active center of the enzyme [86]. The removal of DPCs via TDP1 requires the partial degradation of the DPC by a proteasome [37,87,88,89] and the subsequent processing of the DNA backbone by polynucleotide-3′-phosphatase (PKNP) and canonical repair pathways for the re-ligation of the backbone [90]. Recruitment of TDP1 is achieved by PARylation, implicating an interaction of TDP1 and PARP1 that is also involved in the recruitment of downstream repair factors like XRCC1 [91,92]. This links the function of TDP1 with the mechanism of base excision repair (BER) [93,94]. The complexity of DPC repair is reflected in the CPT hypersensitivity of yeast *tdp1* mutants, which is only detectable in the absence of at least one further repair enzyme [67,72,95,96].

The importance of TDP1 for genome stability is further highlighted by the occurrence of the human autosomal recessive inheritable syndrome SCAN1 (spinocerebrellar ataxia with axonal neuropathy) by homozygous mutations of the *TDP1* gene. This neurodegenerative disease leads to a dieback of neurons of the cerebellum and spinal marrow, thereby causing musculoskeletal system disturbance [97,98].

Similar to the activity of TDP1 at TOP1ccs, tyrosyl-DNA phosphodiesterase 2 (TDP2) is crucial for the hydrolysis of 5′-tyrosyl-phosphodiester bonds at stabilized TOP2-DNA intermediates (TOP2ccs). In doing so, TDP2 promotes a crucial step for the repair of this specific type of enzymatic DPC, which is located adjacent to a DSB [99,100]. With the exception of *Medicago truncatula* [101], plant TDP2 homologues remain poorly characterized so far.

Although several DPC repair strategies rely on proteolytic activity for the efficient removal of covalent DNA-protein adducts, the main pathway based on degradation of the protein moiety has only recently been discovered [72]. In yeast, a central role in the repair of enzymatic (as well as non-enzymatic) DPCs could be assigned to the metalloprotease Wss1 (weak suppressor of smt3 protein 1) [72]. Wss1 was already identified in 2001 and was firstly connected to the SUMO pathway [102,103,104]. While Wss1-deficient yeast lines exhibited hypersensitivity to formaldehyde, a synergistic hypersensitive effect was detected for ∆*wss1* ∆*tdp1* after TOP1cc induction via CPT treatment. Rescue of the severe growth defects in the double mutant via additional deletion of TOP1 clearly indicate that TDP1 and Wss1 are involved in the repair of TOP1ccs using parallel pathways [72]. Wss1 is also involved in the repair of formaldehyde-induced DPCs, due to the lack of the specific tyrosyl-phosphodiester bonds that are repaired by TDP1. The protease function of Wss1 has been shown to be crucial for its role in DPC repair, as complementation analyses of ∆*wss1* ∆*tdp1* lines with a Wss1 version containing a mutated active center of the protease domain could not rescue their hypersensitive phenotype [72]. As a protease, Wss1 is able to target a much broader group of targets compared to TDP1. After Wss1-mediated proteolytic degradation of the protein, the small remaining peptide is now accessible for further downstream repair mechanisms such as translesion synthesis, which involves damage-tolerant translesion polymerases [32]. These findings are supported by the detection of a Wss1-dependent mutagenesis after formaldehyde treatment, where Wss1-deficient lines exhibited a reduced mutagenesis rate compared to the wildtype [72]. Additionally, it has been shown that the metalloprotease acts mainly during the replicative phase of the cell cycle and enables the complete replication of DPC-containing DNA. Consequently, Wss1 highly contributes to the maintenance of genomic integrity.

Based on structural similarities of the zinc-metalloprotease domain, the protein SPRTN (SprT-like N-terminal domain, also known as DVC1) was suggested to be the respective repair protease in mammals [72]. Mutations in Hs*SPRTN* lead to the development of Ruijs-Aalfs syndrome, which is associated with genomic instability, progeroid features and a high susceptibility to the early onset of hepatocellular cancer [105,106].

After treatment of SPRTN-deficient mouse embryo fibroblast cells with formaldehyde, CPT and etoposide hypersensitivity has been detected. Thus, it can be confirmed that SPRTN is indeed the functional mammalian homologue of Wss1 [107,108].

Bioinformatic analyses have hinted to the existence of Wss1/SPRTN-type proteases in the plant kingdom as well. For plants and some fungi, a second Wss1 homologue, Wss1-UBL (later called WSS1B), was identified, which is characterized by the eponymous N-terminal ubiquitin-like (UBL) domain [32].

To check whether the pathway for DPC repair via proteolytic degradation is conserved in plants, Cas9-generated Arabidopsis mutant lines of At*WSS1A* (Wss1) and At*WSS1B* (Wss1-UBL) have been characterized, and no indication of AtWSS1B in DPC repair was found. In contrast, WSS1A has been identified as a crucial factor in the repair of both enzymatic DPCs as a result of CPT as well as cis-platin-induced non-enzymatic DPCs [109]. Further epistasis analysis revealed more insight into plant DPC repair. The analysis of At*tdp1* At*wss1A* double-mutant lines revealed a synergistic hypersensitivity after treatment with CPT, but not cis-platin, while the *tdp1* single-mutant line did not show any hypersensitivity. WSS1A and TDP1 consequently act in parallel pathways in the repair of enzymatic TOP1ccs, although WSS1A is the more significant factor. In line with the enzymatic function of TDP1, no role in the repair of non-enzymatic DPCs (which do not harbor any tyrosyl-phosphodiester bonds) was detected.

**Figure 3:**
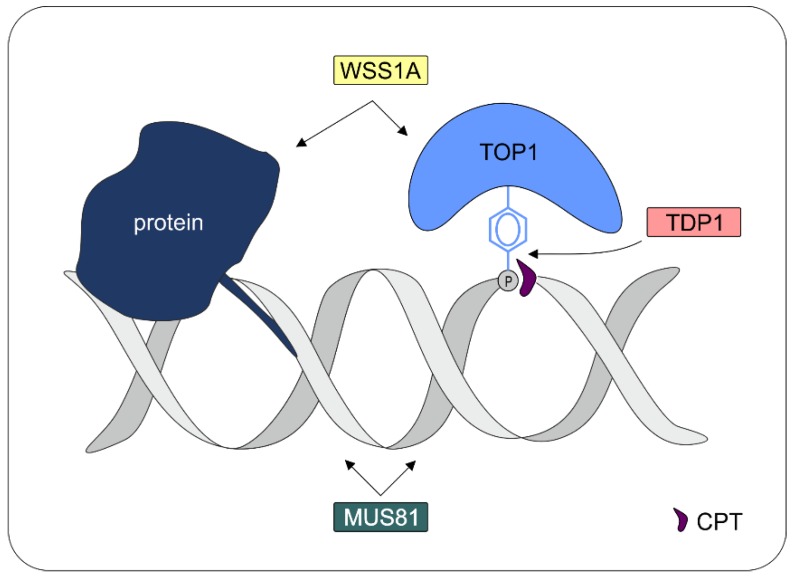
Repair of DPCs in *Arabidopsis thaliana*. DPCs in general can be repaired via proteolytic degradation by WSS1A and in a parallel pathway via endonucleolytic cleavage by MUS81. Enzymatic DPCs like CPT-induced TOP1ccs can be additionally repaired via hydrolysis of the tyrosyl-phosphodiester bond by TDP1.

The structure-specific endonuclease MUS81 is of special importance in plants, acting as a key player in DNA repair [110]. Biochemical analysis has demonstrated the involvement of AtMUS81 in a complex with its interacting partner AtEME1A or AtEME1B in the dissolution of 3′ flaps and nicked Holliday junctions, as well as at a minor rate for intact Holliday junctions [111]. Indeed, an important role for AtMUS81 could be revealed in epistasis analysis, demonstrating the involvement of the endonuclease in the repair of enzymatic as well as non-enzymatic DPCs via a third and predominant pathway in parallel to the protease WSS1A and the phosphodiesterase TDP1 [109]. Consequently, at least three independent pathways for DPC repair exist in Arabidopsis. The first pathway nucleolytically targets the DNA via the endonuclease MUS81 (at enzymatic and non-enzymatic DPCs). The second pathway proteolytically degrades the proteinaceous part of enzymatic and non-enzymatic DPCs via WSS1A, whereas the third pathway enzymatically hydrolyses the tyrosyl-phosphodiester bond of trapped topoisomerase 1 via TDP1 (Figure 3).

## 5. Repair of Intrastrand CLs and ICLs

Intrastrand CLs compromise only one DNA strand, thus leaving the other available as a template. This enables repair either via NER or during replication bypasses via the pathway of postreplicative repair (PRR) [112,113]. For UV-induced intrastrand CLs such as pyrimidine dimers, most organisms, including plants, possess the additional possibility for repair via specialized enzymes called photolyases. In the process of photoreactivation, these enzymes are able to revert the covalent bond of base dimers in a light-dependent manner [114].

ICL repair is of particular complexity, as both DNA strands are affected by the lesion and therefore repair is lacking a sound template. In mammals, the main mechanism for ICL repair is the so-called Fanconi anemia (FA) pathway that involves 22 *FANC* (Fanconi anemia complementation group) genes (*FANCA/ B/ C/ D1/ D2/ E/ F/ G/ I/ J/ L/ M/ N/ O/ P/ Q/ R/ S/ T/ U/ V* and *W*) [115,116,117,118,119]. In humans, germline mutations in these genes lead to the rare autosomal recessive disease Fanconi anemia, which is associated with severe bone marrow failure, chromosomal breakage and innate physical malformations [116,120].

In the Fanconi anemia pathway, ICLs are recognized via a complex composed of the helicase FANCM, FA-associated protein 24 (FAAP24) and MHF. The loading of the core complex (involving 10 FANC proteins, 3 FAAPs and MHF1/2) lead to the monoubiquitinylation of FANCI and FANCD2 (ID complex) [116,121,122,123]. The ID complex then recruits DNA endonucleases like MUS81, SLX1, and XPF/ERCC4/FANCQ, thereby accomplishing the unhooking of the CL by cutting adjacent to the ICL [123]. During this process, a DNA adduct persists on one strand while a break occurs on the other strand. The DNA adduct can further be bypassed via translesion synthesis and afterwards be eliminated via NER. The DSB is then repaired via HR [123,124]. In general, ICLs are repaired differently depending on the cell cycle phase. In the G1-phase, ICLs can be repaired in a recombination-independent manner by unhooking the CL via endonucleolytic cleavage. Afterwards, translesion synthesis can take over, synthesizing the sequence gap with the help of error-tolerant translesion polymerases. In the next step, the CL, which is merely attached to one DNA strand, can be excised via NER followed by repair synthesis.

If a covalent linkage of the complementary DNA strands occurs during the replicative phase of the cell cycle, the repair involves an additional step, as a one-sided DSB arises. In the recombination-dependent repair, unhooking of the CL occurs as before, followed by translesion synthesis (TLS) and NER. The DSB generated by the unhooking is subsequently repaired via HR and the replication fork gets restored after resolution of the recombination intermediates [125,126].

In plants, only around half of the 22 known mammalian *FANC* genes are conserved: *FANCD1 (BRCA2)/ D2/ E/ I/ J (BRIP1)/ L/ M/ O (RAD51C)/ Q (ERCC4)/ R* and *T [127]* (Table 1). However, efforts towards the elucidation of the specific network for plant ICL repair have, to date, only successfully linked two of the conserved *FANC* genes (the helicases FANCJ and FANCM) to ICL repair, indicating that there is no classical FA pathway in plants [128,129,130,131]. Nevertheless, in recent years a multitude of ICL repair factors have been identified in plants, shedding light on this complex mechanism.

The helicase FANCJ, also known as BACH1 (BRCA1-associated C-terminal helicase 1) or BRIP1 (BRCA1 interacting protein), has multifunctional roles in the maintenance of genome stability [141]. In Arabidopsis, two *FANCJ* homologues exist, *FANCJA* and *FANCJB*. Although the two AtFANCJ proteins are 66.2% identical to each other, only FANCJB has been demonstrated to play a role in ICL repair. This is reflected by the hypersensitivity of the respective mutants towards MMC treatment [129].

In addition to the conserved *FANC* genes, homologues of Fanconi anemia-associated proteins such as FAN1 (Fanconi/FANCD2 associated nuclease 1) and MHF1 [131,142] have been identified in Arabidopsis, and both proteins play a role in ICL repair. The nuclease FAN1 is not conserved in all eukaryotes, but an essential function in human ICL repair has been demonstrated [143]. Arabidopsis FAN1 is involved in ICL repair and, interestingly, both its nuclease and ubiquitin-binding zinc finger domain are essential for this function [142]. The histone fold-containing protein AtMHF1 is involved in a common pathway with the FA helicase FANCM, acting in parallel to the RecQ helicase RECQ4A [131]. Astonishingly, FANCM, which is essential for ICL recognition and one of the central components of the FA core complex in humans, appears to fulfil only a minor function in plants. FANCM-deficient Arabidopsis plants do not depict MMC hypersensitivity, and the involvement of FANCM in ICL repair is only revealed when additional repair factors from parallel pathways are missing, such as RECQ4A [131]. Although most *FANC* genes in plants do not possess a conserved role in ICL repair, some are nevertheless important to the maintenance of genome stability in different ways. For example, AtFANCD2 and AtFANCM have been shown to fulfil important roles in meiotic recombination [128,134,137].

RTEL1 is a Fe-S cluster helicase closely related to FANCJ. RTEL1 is a conserved key factor in the preservation of telomere stability, promoted by its ability to dissolve T-loops and G4 structures [144,145]. As double-mutant lines of the *RTEL1* and *FANCJ* homologues in *Caenorhabditis elegans* are synthetically lethal, both helicases were suggested to carry out essential functions in an independent manner [146]. For AtRTEL1, besides an antirecombinogenic function, an involvement in ICL repair has been shown [147,148] whereby the helicase acts in parallel to both FA helicases FANCM and FANCJB [129,147]. Moreover, a crucial role in the maintenance of 45S rDNA repeats was shown for RTEL1, thereby acting independently of the FA helicase FANCJ and the RTR-complex partner RMI2 [147,149].

NER is a central component of DPC and ICL repair in mammals, and a conserved involvement of NER in plant ICL repair seems likely, as mutants and RNAi lines of the plant *XPF* homolog *RAD1* depict strong MMC hypersensitivity [113,150]. The involvement of NHEJ (KU70/80, XRCC4, LIG4) and MMEJ (TEB) factors in the repair of MMC-induced lesions are most likely based on the occurrence of DSBs during replication-dependent repair of ICLs [113,151,152]. In such a scenario, HR-dependent repair mechanisms also participate as RAD51 homologs, and BRCA1 (including interaction partners) have been identified as ICL repair factors in plants [152,153,154,155].

**Figure 4:**
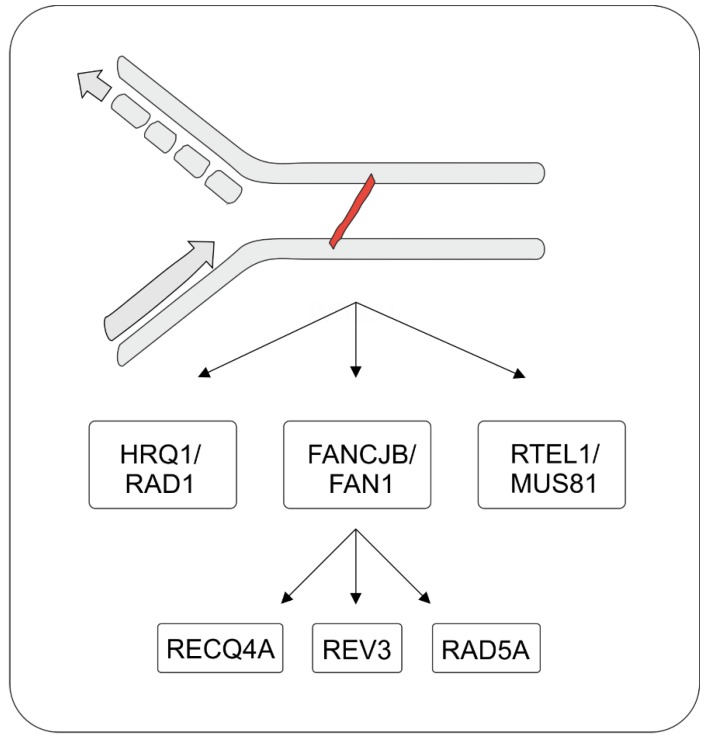
Model for the ICL repair network in *Arabidopsis thaliana*. The initiation of ICL repair is based on the activity of helicases and nucleases, which define three independent pathways. The helicase FANCJB acts together with the nuclease FAN1 in ICL repair. After initial processing, the repair is completed via RECQ4A, REV3 or RAD5A. A second parallel pathway is defined by the helicase HRQ1 in combination with the endonuclease RAD1, while the third pathway is dependent on the helicase RTEL1 and the endonuclease MUS81.

Initially, a three-branched model was proposed for plant ICL repair that was defined by the nuclease MUS81, the helicase RECQ4A and the translocase RAD5A [156]. Within the last nine years of crosslink research, further CL repair factors have been integrated in the model in agreement with the initial findings. The latest studies in *Arabidopsis thaliana* now propose a model (Figure 4) for ICL repair initiation in plants that is mediated by two main repair branches, with are both defined by the interaction of an Fe-S cluster helicase with a nuclease.

We assume the involvement of the following enzymes in the initial steps of the repair pathways: the helicase FANCJB and the nuclease FAN1 representing one branch, acting independently of the helicase RTEL1 and the endonuclease MUS81 [129,147]. The nucleases might be involved in the unhooking step of ICL repair, which is achieved by incisions into the DNA followed by an unwinding of the damaged DNA by the respective helicase. Downstream of FANCJB/FAN1, epistasis analysis has revealed that ICLs can further be processed by at least three different subpathways [129,142,156,157]. The first repair pathway is based on the Arabidopsis homologue of the human BLM helicase, RECQ4A. RECQ4A has been shown to be part of the RTR complex, as a key player in the dissolution of recombination intermediates, and it also plays a role in ICL repair independently of MUS81 [156,158]. The involvement of RECQ4A in a parallel pathway to MUS81 is further supported by the fact that double mutants of *mus81* and members of the RTR complex lead to synthetic lethality [110,159]. Furthermore, a role in ICL repair has been demonstrated for the RTR complex partner TOP3α, hinting to a joint involvement of the complex [159]. Interestingly, a RecQ-like helicase HRQ1, which is proposed to be the yeast and plant homologue to human RECQ4, was recently demonstrated to act in ICL repair in Arabidopsis wherein a role parallel to RECQ4A and similar to that of RAD5A was revealed [160]. Furthermore, HRQ1 and RAD1 were proposed to cooperate in ICL repair, indicating the possibility of another helicase nuclease association for the initial repair steps. The second and third subpathways are defined by the two branches of PRR, mediated by the translocase RAD5A and the catalytic subunit of the translesion polymerase zeta, REV3 [113,129,156,157]. AtRAD5A is a homologue of yeast Rad5, acting in the error-free pathway of PRR [113]. Thereby, RAD5A fulfils a dual role, both mechanistically as translocase, and regulatory in the polyubiquitination of PCNA. In complementation analyses, both enzyme activities has been shown to be necessary for ICL repair in Arabidopsis [113]. The RAD5A translocase has further been classified in ICL to act independently of AtRAD1-mediated NER and AtTEB-mediated MMEJ. REV3, in contrast, is involved in the error-prone mechanism of PRR, called TLS [157]. Presumably, repair intermediates of ICLs in Arabidopsis can be processed either via the two parallel pathways of PRR or via the RecQ helicase RECQ4A following the unhooking of the ICL.

Due to the different properties of CLs, the repair network for intrastrand CLs differs from that of ICLs. So far, no elaborate model has been developed in plants, but a multitude of repair factors have been identified in Arabidopsis, with most of them playing a role in the repair of both types of CL, albeit in different contexts. The basic three-branched model, mediated by the RecQ helicase RECQ4A, the nuclease MUS81 and the PRR translocase RAD5A, seems to also apply to intrastrand CL repair [156]. Furthermore, a prominent role was proposed for the RECQ4A-associated RTR complex, as all members (RECQ4A, TOP3α, RMI1, RMI2) were shown to cooperate in intrastrand CL repair [83,149,158]. As such, a conserved function in plants seems likely, as a function for the *P. patens* RECQ4 homolog in DNA repair was recently confirmed [161]. This could also be linked to the function of the RTR complex in HR, which is a further important mechanism for intrastrand CL repair as multiple RAD51 homologs and RAD54 were shown to be involved in intrastrand CL repair in Arabidopsis [138,162,163,164]. However, in general, a number of factors cooperating in ICL repair in plants do not seem to do so in intrastrand CL repair. Although the Fe-S cluster helicase RTEL1 defines an ICL repair pathway together with MUS81, this is not the case for intrastrand CLs, where both factors act independently [147]. In the same study, a hidden role for the FA helicase FANCM was also defined as acting in parallel to RTEL1. Furthermore, a role in intrastrand CL repair was confirmed for the FA-associated nuclease FAN1 [160]. PRR is an important mechanism for the repair of intrastrand CLs, reflecting the importance of both RAD5A and REV3 in Arabidopsis [157,165]. Similar to its involvement in ICL repair, RAD5A was demonstrated to act independently of RAD1-mediated NER and TEB-dependent MMEJ [113]. Error-prone PRR mediated by REV3 also fulfils an independent role in parallel to RECQ4A, MUS81 and RAD5A [157]. A factor that might unite the different branches of intrastrand CL repair is the RecQ-like helicase HRQ1, which cooperates with RAD1, RECQ4A, RAD5A and FAN1, leaving only the backup endonuclease MUS81 in a separate pathway [160].

## 6. Conclusions and Perspectives

Crosslinks of all described types are toxic lesions, strongly threatening the genomic integrity of the cell. Therefore, efficient repair strategies are indispensable for cell viability. While the repair mechanisms of intrastrand CLs and ICLs have been studied for decades, research on DPCs has only been sparsely conducted. With the identification of DPC-processing proteases in 2014 [72], DPC repair mechanisms have been receiving more attention and now represent a quickly developing scientific field. As DPC repair mechanisms are currently only partially elucidated, it will be interesting to investigate the interplay of different repair pathways in respect to DPCs in plants in the future.

Although it might not seem obvious at first glance, a better understanding of CL repair mechanisms could also help in fighting the obstacles of climate change in agriculture. In future, plants insufficiently adapted to heat and salt stress might produce a surplus of stress-induced ROSs, resulting in more DNA damage. Also, as cultivation of plants at higher altitudes surges, plants will be exposed to higher doses of UV light, threatening both genome stability and ultimately leading to reduced yields. Thus, further research on CL repair mechanisms in plants could help ensure food security in an uncertain future.

## Figures and Tables

**Table 1:** *FANC* genes in plants. The table gives an overview of the conserved *FANC* genes in plants, the synonymous nomenclature where present, keywords and their functions and respective references.

*FANC* Gene	Synonym	Keywords	Reference
*FANCD1*	*BRCA2*	Somatic and meiotic HR	[132,133]
*FANCD2*		Somatic and meiotic HR	[134]
*FANCE*		No role in meiotic HR	[135]
*FANCI*		No role in meiotic HR	[135]
*FANCJ*	*BRIP1,* *BACH1*	Helicase, ICL repair, replicative repair, rDNA stability	[129]
*FANCL*		Ubiquitin ligase, no role in meiotic HR	[135,136]
*FANCM*		Helicase, Antirecombinase, ICL repair, suppression of somatic and meiotic HR	[128,137]
*FANCO*	*RAD51C*	Recombinase, Mitotic and meiotic HR	[138]
*FANCQ*	*ERCC4, RAD1, UVH1*	Endonuclease, NER	[112,139]
*FANCR*	*RAD51*	Recombinase, mitotic and meiotic HR	[133,140]
*FANCT*		uncharacterized	[127]

## Abbreviations

BER	Base excision repair
CL	Crosslink
CPT	Camptothecin
DPC	DNA-protein crosslink
DSB	Double-strand break
Eto	Etoposide
FANC	Fanconi anemia complementation group
HR	Homologous recombination
ICL	Interstrand crosslink
IR	Ionizing radiation
MMC	Mitomycin C
MMEJ	Micro-homology mediated end-joining
NER	Nucleotide excision repair
PRR	Post replicative repair
ROS	Reactive oxygen species
TDP1/2	Tyrosyl-DNA-phosphodiesterase 1/2
TLS	Translesion synthesis
TOP1cc	Topoisomerase 1 cleavage complex
ZEB	Zebularine
